# Experiences of increased food insecurity, economic and psychological distress during the COVID-19 pandemic among Supplemental Nutrition Assistance Program-enrolled food pantry clients

**DOI:** 10.1017/S1368980021004717

**Published:** 2021-12-06

**Authors:** Robin T Higashi, Anubha Sood, Ana Belen Conrado, Kathryn L Shahan, Tammy Leonard, Sandi L Pruitt

**Affiliations:** 1 University of Texas Southwestern Medical Center, 5323 Harry Hines Blvd, Dallas, TX 75390-8557, USA; 2 Harold C. Simmons Comprehensive Cancer Center, 2201 Inwood Road, Dallas, TX 75390, USA; 3 University of Texas Health Science Center, School of Public Health, Houston, TX, USA; 4 University of Dallas, Department of Economics, Irving, TX, USA

**Keywords:** Coronavirus, Hunger, Health impact, Food bank, Food pantry, Qualitative

## Abstract

**Objective:**

The COVID-19 pandemic initially doubled the rates of food insecurity across the USA and tripled rates among households with children. Despite the association among food insecurity, chronic disease and psychological distress, narratives depicting the experiences of already food insecure populations are notably underrepresented in the literature. The current study assessed the impact of COVID-19 on clients of a food pantry who were also enrolled in the Supplemental Nutrition Assistance Program (SNAP).

**Design:**

A qualitative study probing the effects of the pandemic on daily living, food needs, food buying and food insecurity. Interview transcripts were analysed using a combined deductive and inductive approach.

**Setting:**

Interviews were conducted via telephone between May and June of 2020.

**Participants:**

Equal numbers of English- and Spanish-speaking clients (*n* 40 total).

**Results:**

Three main findings emerged: (1) the pandemic increased economic distress, such as from job loss or increased utility bills due to sustained home occupancy and (2) the pandemic increased food needs, food prices and food shortages. In combination with economic stressors, this led to greater food insecurity; (3) increased economic stress and food insecurity contributed to increased psychological stress, such as from fear of infection, isolation and children being confined at home.

**Conclusions:**

Despite federal legislation and state and local programmes to alleviate food insecurity, COVID-19 exacerbated economic hardship, food insecurity and psychological distress among urban SNAP and food pantry clients. Additional research is needed to identify the most effective policies and programmes to ameliorate the short- and long-term health and economic inequities exacerbated by the pandemic.

In the early months of the COVID-19 pandemic, the rates of food insecurity doubled across the USA and tripled among households with children^(1)^. Texans experienced some of the worst rates of food insecurity nationwide, with one report estimating that more than one in four households (26·8 %) experienced food insecurity during April–May 2020^(1,2)^. Some households experiencing food insecurity, defined as lack of consistent access to adequate nutritious foods^(3)^, rely on food pantries and/or the Supplemental Nutrition Assistance Program (SNAP), formerly known as ‘food stamps’^(4)^. Demand for food from the North Texas Food Bank, the largest distributor to food pantries in the Dallas Fort Worth metroplex, increased 60 % from 2019 to 2020, with over half of families seeking food assistance from food pantries for the first time^(5)^. And across the USA, first-time SNAP enrollment increased by unprecedented numbers during the COVID-19 pandemic^(6,7)^.

Studies focusing on the impact of COVID-19 on populations who were food insecure before the pandemic have mixed findings. Siddiqi et al. found that food insecurity increased significantly among participants enrolled in SNAP before and after the pandemic^(8)^, whereas Molitor et al. reported that new federal benefits available in response to COVID-19 may have reduced very low food security among low-income households before and shortly after the economic downturn from the pandemic^(9)^. Feng et al. indicated that racial/ethnic disparities in food insecurity among African American and Hispanic/Latinx populations were exacerbated by the pandemic^(10)^, whereas Dubowitz et al. concluded that disparities had not worsened, but rather barriers differed among racial/ethnic groups^(11)^. A large and growing body of literature has also assessed characteristics of the people experiencing food insecurity during COVID-19 without distinguishing whether participants were enrolled in SNAP prior to the pandemic^(11–15)^. These studies documented that African American and Hispanic/Latinx populations were especially affected by the pandemic^(11)^ and that SNAP enrollment was associated with reduced food insecurity during the pandemic^(15)^. Further, studies confirmed that food insecurity during the pandemic, as before the pandemic, was associated with depression, anxiety and stress^(14)^.

Fewer studies have examined the health impacts of COVID-related food insecurity among existing SNAP and food pantry clients. This area of research merits further attention because individuals from households receiving both types of food assistance have poorer health compared with those receiving SNAP or charitable food assistance alone^(16)^. Studies have shown that, even before the pandemic, SNAP benefits alone were insufficient in alleviating food insecurity for many households^(17–19)^. Given the documented association between food insecurity, chronic disease and psychological distress^(20–24)^, it seems likely the pandemic would exacerbate negative health consequences for already food insecure populations^(25,26)^. Importantly, however, qualitative studies documenting the health and economic experiences of individuals and families experiencing food insecurity in the USA during the COVID-19 pandemic are notably absent. It is critical that these narratives contribute to the body of evidence about the impact of COVID-19 in order to both convey and humanise the perceptions and experiences of affected individuals. Furthermore, these interviews, completed in just two months, were able to capture and portray the sudden and significant ‘shocks’ caused by the pandemic during a unique period of time.

This paper contributes empirical evidence of the effects of the COVID-19 pandemic on the economic and food security status, food purchasing behaviours and psychological health of urban food pantry clients who were also enrolled in SNAP.

## Methods

We conducted a qualitative study consisting of semi-structured interviews with individuals enrolled in SNAP who were also clients of Crossroads Community Services (Crossroads), a Dallas, Texas area food pantry. Participants in this qualitative study were identified from a larger cohort of clients participating in a randomised control trial (RCT)^(27)^. The trial, briefly, had two arms, and clients were randomly assigned to a scheduled food pantry appointment each month for seven months: either early in the month (close in time to the clients’ SNAP benefit replenishment) or later in the month (approximately two weeks after SNAP benefit replenishment)^(27,28)^. Initially, the goal of the qualitative study was to assess clients’ experiences with the scheduled appointments. However, because qualitative data collection occurred soon after the start of the pandemic, we altered the interview guide to add additional questions probing the impact of the pandemic on food insecurity and food-related behaviours.

### Setting

Crossroads Community Services is the largest food distributor and member agency of the North Texas Food Bank, providing food to clients through its network of 140 community distribution partners in addition to its in-house pantry in Dallas. Clients register by providing income documentation every six months to confirm household eligibility for assistance at < 185 % of the federal poverty level. The median monthly household income of Crossroads clients in 2020 was $1235; with an average household size of four, this income level is approximately 50 % of the federal poverty guidelines. Of Crossroads clients, 66 % are Hispanic/Latinx and 28 % are African American.

### Sampling

We sampled from a pool of 109 food pantry clients participating in the RCT based primarily on two characteristics: (1) timing of food pantry appointment (equal proportion of early- and late-month appointments), because this may have affected experiences of food insecurity and (2) preferred language (equal proportion of English- and Spanish-speakers), as these populations may have had different pandemic-related experiences. Participants were also chosen to ensure our sample varied in regard to household size and dollar amount of the households’ monthly SNAP assistance amount. Participants who opted-out of interviews at the time they enrolled in the RCT were excluded.

### Recruitment

Interviewers telephoned potential participants who had completed at least six monthly appointments at Crossroads, as we believed this was an adequate time frame from which participants could comment on their experiences in the RCT. Clients provided informed verbal consent for participation in audio-recorded interviews and were provided with a $20 gift card in appreciation for their time.

### Data collection

Three study team members with qualitative expertise (RTH, AS, ABC) conducted semi-structured interviews on the telephone in English or Spanish. By employing semi-structured interviews, we were able to allow participants to elaborate on their responses beyond the originally proposed, *a priori* questions listed in the interview guide (see Appendix A). This was important because, given the rapid development of events during the early months of the pandemic, we were not able to pre-test interview questions and obtain stakeholder feedback. Importantly, many of the findings that emerged during data collection were reported spontaneously or in response to follow-up probes rather than as the result of the initial interview questions. Each interview was audio-recorded with the client’s permission and probed topics including the effects of the pandemic on daily living and food needs, food purchasing habits and experiences with food insecurity.

### Data analysis

Audio-recorded interviews were transcribed and de-identified and thematically analysed using NVivo 12·0 (QSR Australia). Using a deductively driven codebook corresponding to the interview guide, three team members met weekly to discuss, jointly code and refine codebook definitions for the first 30 % of transcripts. We also included an ‘emergent’ code to categorise themes emerging during the coding process that were not directly anticipated by the original interview questions (e.g. about food insecurity) but represented important findings (e.g. about psychological impacts). The codebook was then finalised, and two team members (AS, ABC) double-coded the remaining transcripts, meeting weekly to resolve coding discrepancies. Once all transcripts were coded, the two team members synthesised findings from each theme in a summary report. All summary reports were reviewed by the senior qualitative investigator (RTH) for quality and cohesion. Findings and representative quotes were interpreted with the entire research team. Findings reported here predominantly derive from data coded under the labels ‘coronavirus’, ‘food insecurity’ and/or ‘emergent’.

## Results

### Participants and study context

We conducted *n* 40 semi-structured interviews, each lasting an average of 26 min (range 17–41 min). Table 1 includes participant characteristics collected by Crossroads during the RCT enrollment (October 2019–January 2020) and food security and self-rated health status collected at RCT follow-up (June 2020–July 2020). Clients were predominantly Hispanic/Latina women, and over half experienced low or very low food security, as measured using the U.S. Department of Agriculture Adult Food Security Survey Module^(29)^. Interviews were conducted during the months of May–June of 2020, approximately 2–3 months from the start of the COVID-19 pandemic.

**Table 1:** Participant socio-demographic characteristics

	*n*	%
Preferred language		
English	20	50
Spanish	20	50
Race/ethnicity		
Hispanic/Latinx	30	75·0
African American	9	22·5
White	1	2·5
Sex		
Female	39	97·5
Male	1	2·5
Household size (# persons)		
Average	5·6	
Range	1–10	
SNAP monthly benefit amount ($)		
Average (mean)	389	
Range	15–1500	
Food security*		
High	3	7·5
Marginal	6	15·0
Low	3	7·5
Very Low	28	70·0
Self-Reported Health		
Excellent	3	7·5
Very good	3	7·5
Good	24	60·0
Fair	10	25·0
Poor	0	0

* Measured using the U.S. Department of Agriculture (USDA) Adult Food Security Survey Module^(29)^.

**Fig. 1:**
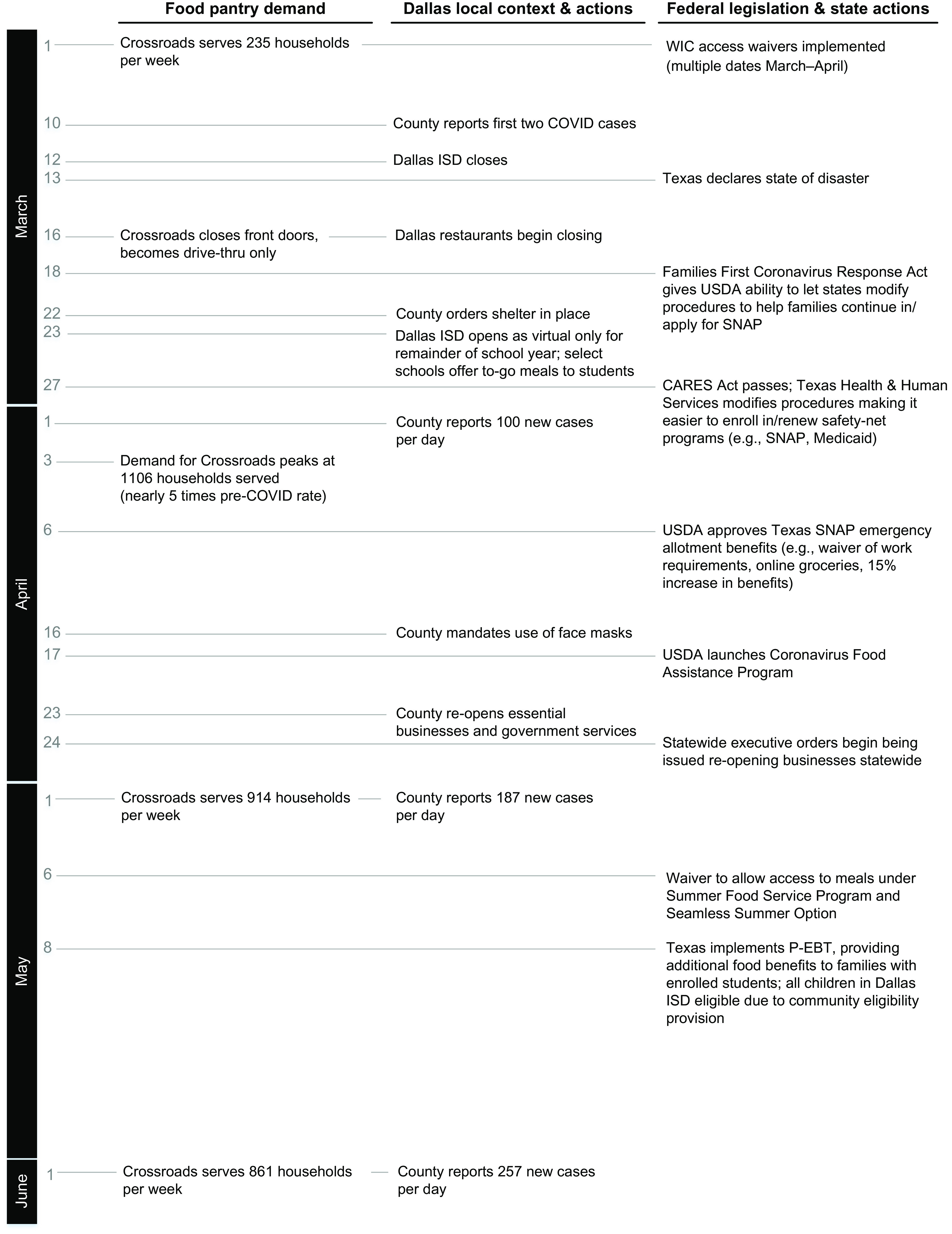
Multilevel factors affecting the experiences of Dallas-area participants experiencing food insecurity in early 2020

**Fig. 2:**
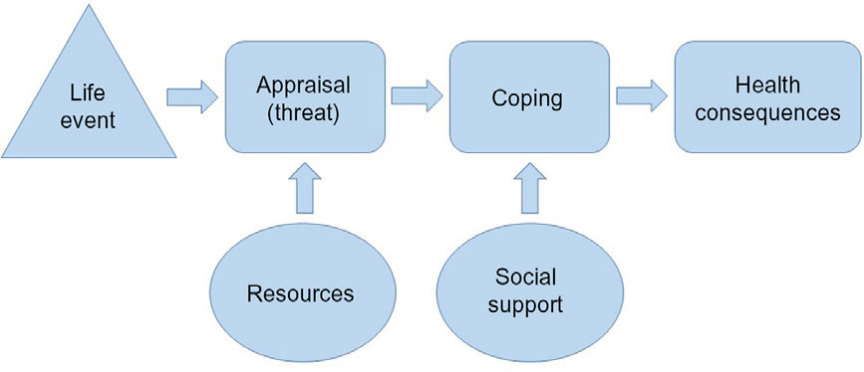
Transactional Theory of Stress

Figure 1 illustrates a timeline of factors at multiple, interrelated levels that shaped the experiences of our participants during and immediately prior to the study period. These factors help contextualise the reported experiences of participants during this unique time characterised in part by rapidly changing programmes and policies implemented in response to the growing scale of the pandemic. The socio-ecological^(30)^ framework posits that spheres of influence at multiple levels interact in complex ways to influence health and wellbeing. As depicted in the figure, organisational factors (e.g. food pantry demand), community factors (e.g. school district closures) and the rapidly shifting federal and state policy landscape influenced our study environment. During our study period, for example, as the number of COVID-19 cases increased, schools and workplaces closed. In response to the resulting economic downturn, a patchwork of federal, state and local policies were implemented to alleviate food insecurity; some of these are reflected in Fig. 2 ^(31–36)^. Notably, several policies went into effect that improved access to food assistance for our participants; for example, SNAP renewals were automated, and SNAP benefits were increased.

In our analysis of interview transcripts, we identified three key themes among participant experiences during the first year of the pandemic: (1) the pandemic increased economic hardship; (2) the pandemic increased food needs, leading to greater food insecurity and (3) the pandemic increased psychological distress. We use the Transactional Theory of Stress^(37)^ as a framework to illustrate the relationship among each of these themes (Fig. 2). In this model, a life event leads to a threat or stress appraisal, which is moderated by resources. Coping, which is moderated by social support, follows the appraisal, which in the end impacts one’s health outcomes. As participant narratives indicate below, the pandemic increased economic hardship (i.e. fewer resources) and to some extent increased social isolation (i.e. less social support), which increased stress and had implications for participants’ health outcomes.

### Theme 1: The pandemic increased economic hardship

Economic hardship was exacerbated by two related factors: diminished household income and increased home expenses.

Diminished family income resulted from business closures and employers and/or public health authorities limiting opportunities for employment during the pandemic. Family members either lost jobs altogether or their working hours were cut drastically. COVID-related underemployment adversely affected participants’ ability to procure food and increased their reliance on the food pantry.

One participant narrated her distress at not being able to afford food for her children due to job loss.

*‘Interviewer: Have you ever had a time where you didn’t have enough money to buy the food you needed?*

*Participant: It happened about twice.*

*Interviewer: Can you tell me about that?*

*Participant: I didn’t have a job and my children wanted to go to the store to buy something to eat, and I didn’t have enough money.*

*Interviewer: How did you feel?*

*Participant: I cried…because they wanted to eat something different and I didn’t have the money to buy.’ (participant #17, Very low food security, 6 household members)*

A number of participants spoke about how job loss meant that they were strained for money to buy food and were, therefore, relying more on the food pantry.

*‘My husband was working and my daughter was working, and now it’s my husband just working and my daughter doesn’t work. It’s just him now, but since the pantry is still giving me food, it helps me a lot because, at first, we would struggle and I was like, ‘Damn, we’re going to struggle more because we only have one person working.’’ (participant #29, Marginal food security 10 household members)*

Concurrent, in many cases, to reduced income, several participants also reported marked increases in utility payments because numerous household members were socially isolating at home for prolonged periods of time and thus were consuming more electricity, gas and water.

*‘In the summer our bills always go up because everybody, the kids are home all day. But they’re already starting to go up now because they’re home all day long. My son is on the computer doing homework. My daughter is on her school Chromebook doing her work. So lights are on all day.’ (participant #1, Very low food security, 5 household members)*

Regular expenses like food and mortgage or rent also became challenging. As one participant noted, *‘Money has been tighter…our food pantry is almost empty. It’s been bad.’ (participant #3, Very low food security, 3 household members)*. Another noted:

*‘It has affected the payments on the house because my husband works less. He’s working less now and even though Lone Star [card providing access to Texas SNAP program benefits] helps, but there is less work. Like I said, that’s the only good thing, that they’ve given us – more Lone Star.’ (participant #37, Very low food security, 10 household members)*

The ‘Lone Star’ card mentioned by this participant refers to the Electronic Benefit Transfer (EBT) card, similar to a debit card, that allows Texans enrolled in SNAP to spend their benefits at qualified food retailers. Fig. 1 illustrates that Texas SNAP recipients received increased SNAP benefit allotments starting in April 2020.

One participant indicated having to choose between paying bills and having enough to eat. *‘As long as I got a pack of beans and a pack of rice we’ll eat something… If I have to not pay a bill to make sure we eat we’re going to eat.’ (participant #15, Low food security, 5 household members)*

### Theme 2: The pandemic increased food needs, leading to greater food insecurity

Nearly all participants reported that their families’ food needs increased significantly during the pandemic. School closures were a major contributing factor to this increased demand. When schools were not in session, most families could no longer rely on the school’s meal programmes, which added a significant financial burden to already strained household resources. As one participant noted,

*‘My food needs have increased because my sons - they’re here all day now instead of going to school. So before I would make them breakfast and then they would have dinner, but now it’s breakfast, snack, lunch, snack, dinner, snack.’ (participant #15, Low food security, 5 household members)*

Only two participants indicated that they continued to access meals directly from the school during the pandemic. One said,

*‘[At the] school, the lunch that they give us, and then whatever they give me from the Crossroads, it’s been helping me. Now that they’re home, they eat a whole lot more now, because before, they weren’t home. Now they’re just basically eating all day.’ (participant #29, Marginal food security, 10 household members)*

Another commented, *‘My son is hungry all day… All of us here we’re cooking of course a lot more because we’re here all the time.’ (participant #1, Very low food security, 5 household members)*

Families implemented a number of strategies as they struggled to ‘make ends meet’. For instance, many families reported swapping out meal recipe ingredients with cheaper, less nutritious ingredients to cook adequate quantities of food:

*‘I kind of just minimize how I do things or just compromising. Like I said instead of the dumplings I’ll just get the cheap can of biscuits that’s cheaper. I’ve got enough for a 99 cent can of biscuits v. $5·00 to go get those other ingredients to do the dumplings.’ (participant #2, Very low food security, 4 household members)*

A few participants said they borrowed money to get by until their next food disbursement.

*Participant: Yes, or you’ve got to borrow it, the money from someone else, like $20·00, to get at least about two meals, to help me along.*

*Interviewer: You were able to borrow money, you said?*

*Participant: I’ll borrow money and then be able to pay it back. I’ll borrow at most like $20·00 to get it, to get food.*

*Interviewer: Okay. Okay. How often are you not able to make ends meet? Has that been a lot of times recently?*

*Participant: Yes, ma’am. Yes, ma’am. Yep. (participant #5, Very low food security, 3 household members)*

At the same time, a number of participants noted that food prices had risen during the pandemic.

*‘There’s still like, a week where I barely have any food at home and especially now, since all this has started with the Covid-19, I mean, the prices for meat have gotten really expensive. And even other basic things – like, be it rice or sugar or things like that – it’s gone up as well. So, I’m still dealing with not having.’*

*‘Well, this month I ran out of snacks and I have to buy from my pocket… Right now it’s expensive. Like everything is kind of like they raised on the meat and all that. It’s expensive.’ (participant #26, High food security, 8 household members)*

Many participants emphasised how utterly dependent they were on the food pantry to ‘tide them over’, especially after their SNAP benefits ran out.

*‘Basically we didn’t have much. And when we get the stamps [SNAP benefits] we spend it right away. I asked the lady [at the food pantry], I said “Look, everything is appreciated. If you have anything extra to give us to make it through the month that would be awesome.” And she’s like, “If you want to come back in two weeks instead of a whole month you can do that this time.” I said OK. So I think it’s going to be just for this month, these two months.’ (participant #30, Very low food security, 6 household members)*

Two participants reported going to additional food assistance events in order to get by.

*It’s not really a food pantry because they don’t ask for any information and you’re allowed to go daily. They give like, fresh vegetables. I mean, you don’t get to pick what you get but it’s certainly things that it will keep food in my house when I need it. So, I go when I really need something and I don’t have it, I was going to that food place maybe three/four times a week.” (participant #26, High food security, 8 household members)*

### Theme 3: The pandemic increased psychological distress

Participants reported an increase in psychological distress ranging from fear of the virus, managing children at home during school closures, and increased anxiety resulting from changes in household eating behaviours.

One participant described feeling scared about exposing her family to the virus and thus socially isolating in her home.

*‘It has affected everybody greatly. Well, psychologically, because they don’t want to go out and well, at first, I was scared of going out or maybe I would go out, but the children wouldn’t and I might bring a disease in or something like that. So, I think that psychologically, it did affect us.’ (participant #34, Very low food security, 7 household members)*

She went on to describe feeling caught between needing to go out to earn ‘have enough’, but being scared of contracting the virus.

*‘It also affected everybody financially… because my husband didn’t want to go to work sometimes, but now because he didn’t want to, but he was scared. Well, we were all scared, right? So, some days, he didn’t want to go to work and well, I thought, ‘Oh, well, but if you don’t go to work, we won’t have enough.’… So, there were times when someone was sick there where he works, and they were scared and sometimes, they would only work a little bit.’*

Lockdowns and fear restricted the daily routines of many participants, keeping family members socially isolated inside the home more than usual.

*‘Since the pandemic started we’ve not going outside for anything. We don’t leave. I was afraid of taking them outside to the yard, but now we go to the yard to play. But if we see someone walking we go back – I tell them, ‘If you see someone walking come here to the house door and then you can go back when he leaves.’ We’re afraid. And I usually went shopping or to the laundry, but now I can’t go because I have an agreement my husband that we’d not leave both, but only him. So he’s the one who goes to work. He arrives, and if he has to go to the laundry he goes, or during the weekend. And during the weekend he goes to buy what we need. I don’t go outside but to go to the pantry. That’s the only day I leave. And I leave my daughters with my mother-in-law and I go to get the pantry. I come back with my daughters and we stay at home, but we don’t leave for anything else.’ (participant #16, Very low food security, 5 household members)*

Several participants also reported stress relating to managing school-aged children during the COVID-19 lockdown. They expressed difficulty not only in providing adequate meals but also in keeping children entertained and engaged in their schoolwork from home.

*‘Well, it was a little difficult having the five children at home, knowing that they had to do schoolwork, having food at all times because they’re always eating and most of all, helping them deal with all of that too, because they ask, “Why aren’t we going to school?” “Why is this happening?” They get bored. They get mad, and trying to deal with all of that.’ (participant #10, Very low food security, 7 household members)*

For some, pandemic-related fear and anxiety resulted in eating more or at eating at odd hours.

*‘I have a girl who is sometimes eating in the early morning. And I tell her, ‘Why are you awake at this hour, at 1:00 AM eating?’ ‘I’m not sleepy and I’m hungry.’ …I’m concerned about that and I don’t know how to solve it.’ (participant #28, Low food security, 7 household members)*

*‘We’re just eating and eating… I just try to calm my hunger and not eat that much because I know this is because we’re enclosed… This is because I don’t know what to do and I go to open the refrigerator to see what I can find, but I’m not hungry, but anxious because I’m enclosed. But I try to control it.’ (participant #31, High food security, 4 household members)*

## Discussion

We identified increased economic hardship, increased food insecurity and increased psychological distress among a sample of urban food pantry clients who were also enrolled in SNAP at the start of the COVID-19 pandemic. Economic stress from un/under-employment and increased consumption of food and utilities, in combination with school closures, general lockdown restrictions and quarantines led many families to experience deepening food insecurity. Many participants also expressed anxiety and stress resulting from fear of the virus, managing multiple children at home, and food insufficiency. Consistent with the transactional theory of stress, the pandemic increased the ‘threat’ or appraisal of stress by increasing economic strain and food insecurity (i.e. fewer resources) while at the same time reducing coping capacity by decreasing social support. These are valuable contributions to the expanding literature on the links between economic strain and health effects of COVID-19 because they are the first to qualitatively describe the experiences of a population that was already experiencing food insecurity at the start of the pandemic. They demonstrate how the pandemic exacerbated existing stressors and health consequences in an already vulnerable population and provide a snapshot of the experiences and perceptions of this population during a time characterised by rapidly changing circumstances.

Our findings indicate substantial unmet economic hardship and food insecurity in this population at the start of the pandemic. Direct economic assistance, assistance to food banking systems and expansions to SNAP were provided by the federal government via a patchwork of legislative acts in 2020, including The Coronavirus Aid, Relief, and Economic Security (CARES) Act, the Families First Coronavirus Response Act and waivers to enhance access to, continuity of and/or amounts of food assistance programmes such as child nutrition programmes, WIC and SNAP, as shown in Fig. 1. Many studies have documented the close association of need for assistance from programmes such as unemployment, SNAP, WIC and Medicaid during the pandemic^(13,38–40)^. Thus, it is significant that, in the current study, all participants were already receiving SNAP and food pantry assistance prior to the pandemic and their SNAP benefit amount was increased during the pandemic, but nonetheless, many reported food insecurity.

It is also significant that clients experienced increased food insecurity despite initiation of social services and programmatic assistance for families in need. For example, a number of federal, state and municipal policies provided foreclosure and/or eviction moratoria^(41)^; however, economic hardship remained a stressor for many families because there were inadequate resources provided to assist with increased utility costs, food shortages and increased food costs. Additionally, the fact that only two participants (5 %) reported accessing assistance from COVID-19 emergency-funded school meal disbursements suggests that the modified school meal programmes^(42)^ did not effectively substitute for pre-pandemic meals that were provided when children were in school. It is possible that parents were not aware of efforts schools were making to provide food during school closures. It is also possible that transportation constituted a significant barrier for families who previously relied on sending children on the school bus, that they may lack their own transportation or that they may have avoided public transportation given its greater potential risk for exposure to SARS-CoV-2, the virus causing COVID-19. In sum, families who were already food insecure prior to the pandemic suffered increased economic hardship and psychological stress during the onset of the COVID-19 pandemic in spite of legislative and programmatic assistance. This finding is consistent with the transactional theory of stress (Fig. 2), which suggests that stress and health consequences result from an ‘imbalance between demands and resources’ when threats from life events exceed one’s coping abilities^(43)^. In the current study, resource needs outpaced resource availability by exacerbating the economic and psychological stress experienced by already food insecure populations.

One possible explanation for this exacerbation is that COVID-19 created a ‘gradient effect’, magnifying existing inequities and deepening the severity of food insecurity among low-income individuals^(44)^. That is, people living in poverty, or within the margins for income eligibility for SNAP, might be less able to withstand COVID-19-related income shocks or need for increased spending than those not living in poverty before the pandemic. For example, hoarding behaviours by people not living in poverty may leave low-income families without options for food or household staples if they cannot similarly afford to buy in bulk^(45,46)^. Coping is required to moderate stress according to the transactional theory of stress. However, coping behaviours among food insecure populations, such as food sharing among families and neighbors, or strategic food shopping, were riskier or not possible during COVID-19, increasing risks and severity of food insecurity^(46)^. The convergence of food insecurity and risk for COVID-19 is bi-directionally related, with one exacerbating the risk for the other^(25)^. That is, people experiencing food insecurity may face increased risk of exposure to SARS-CoV-2 while attempting to procure food (e.g. using public transportation) and generate income (e.g. working in jobs that cannot be done remotely). At the same time, food insecure populations have higher rates of comorbid conditions and malnutrition that increase risk for severe COVID-19 disease.

These magnified inequities in the experiences of already food insecure populations suggest several policy implications. The 2021 American Rescue Plan Act provided additional, much needed additional assistance for pandemic relief, but sustained and coordinated efforts to increase amounts of SNAP benefits, increase length of enrollment periods for SNAP benefits and address un/under-employment and other pandemic-related economic effects may still be needed^(47)^. For example, participants reported increases in utility payments because of increased home occupancy; assistance with utility payments, as well as rental or mortgage assistance may be required. The need for mental health services greatly increased during the COVID-19 era^(48)^, with depression and anxiety disproportionately affecting women and persons of coloUr^(49)^. At the same time, the availability of mental health providers and the ability to access services are uneven, particularly in states like Texas that did not expand Medicaid^(49)^. Given that food insecurity is associated with depression and anxiety, particularly during the uncertainty and stress of the COVID-19 pandemic^(14,50)^, it is vital that safety net healthcare programmes include affordable and accessible behavioural and mental health coverage^(51)^. Such programmes may be able to address the fear, anxiety, social isolation and ‘stress eating’ behaviours, which are elevated during disaster situations^(37)^ and may negatively affect their health.

The current study is subject to a few limitations. Participants were contacted on behalf of Crossroads, the organisation from which they received monthly food disbursements. Interviewers were employed by an academic institution, not Crossroads, but regardless, the unequal power relationship between interviewers and participants may have affected the way participants described their food procurement behaviours or how they were affected by the pandemic, even though they were informed that their responses would not affect their food allocations. Second, while we believe that many of the same issues of economic hardship, exacerbated food insecurity and psychological distress may also affect communities in smaller urban, suburban and rural areas of the USA, the current study was conducted with a sample of predominantly Hispanic/Latina women in one large urban city in Texas. Third, in order to solicit feedback about their experiences with the RCT, participants recruited for interviews included only those who received six months or more of consistent, ongoing food pantry appointments; thus, we did not capture experiences of clients receiving no or intermittent food pantry assistance.

In sum, the current study contributed qualitative evidence of the health and economic exacerbations from COVID-19 among a sample of urban food pantry clients also enrolled in SNAP early in the pandemic. Narratives from participants raise questions about whether emergency federal and state policies to increase access to SNAP and other programmes were sufficient in meeting the increased economic and food needs of an already food insecure population during the pandemic. One report describing P-EBT program implementation indicated confusion among SNAP participants and an application process that created extensive case-specific communications that burdened applicants and the state^(52)^. This suggests that, while programmes were available, initial challenges with access may have reduced their effectiveness.

While the current study captured economic hardships experienced by participants within three months of the start of the pandemic, policies for SNAP expansion and federal income support did ultimately boost food security rates and economic stability in 2020^(53)^. To inform strategic policy planning, additional research is also required to better understand differences in the health and economic impact of COVID-19 on previously and newly food insecure populations and to assess differences in the unmet needs and experiences among different subsets of these heterogeneous populations. For example, one study found that previously and newly food insecure populations employed different coping strategies during the pandemic crisis, which has important implications for identifying strategies to address mental health and malnutrition in these populations^(54)^. Evaluations of the ongoing impact of the pandemic and relevant programmes on the economic stability, food insecurity and psychological distress of the population will continue to be valuable as the COVID-19 pandemic and its effects persist.

## Supplementary material

For supplementary material accompanying this paper visit https://doi.org/10.1017/S1368980021004717. click here to view supplementary material
