# Supplementary material for: Experiences of increased food insecurity, economic and psychological distress during the COVID-19 pandemic among Supplemental Nutrition Assistance Program-enrolled food pantry clients
Source: Public Health Nutr. 2021 Dec 6;25(4):1027–37. doi: 10.1017/S1368980021004717 (PMC8712963; doi:10.1017/S1368980021004717)
Supplement: Supplementary file 1 [file S1368980021004717sup001.docx]

**APPOINTMENTS**

**CITAS**

Over the past few months, you've been participating in a project where you made appointments to go to the food pantry each month.

Durante los últimos meses, usted ha estado participando en un programa en el cual hizo citas para ir a la despensa de alimentos cada mes.

1. What do you think about the appointments?

¿Qué piensa sobre las citas?

- 1. What do you like about them?

¿Qué le gusta sobre ellas?

- 1. What do you NOT like about them?

¿Qué NO le gusta sobre ellas?

1. Tell me about scheduling your appointments.

Dígame sobre la programación de sus citas.

- 1. Were you able to select a time that was convenient for you?

¿Pudo seleccionar una hora que fuera conveniente para usted?

- 1. Was there anything in particular that made it hard to keep your appointment?

¿Hubo algo en particular que le hizo difícil mantener su cita?

- 1. Do you plan to keep making appointments?

¿Tiene planes de seguir programando citas?

1. What suggestions do you have for how the food pantry could make it easier for you to make and keep your appointments?

¿Qué sugerencias tiene para que la despensa de alimentos pueda hacer más fácil para usted programar y mantener sus citas?

1. Do you think other clients would like to go to the food pantry with appointments? Why or why not?

¿Piensa que a otros clientes les gustaría ir a la despensa de alimentos con citas? ¿Por qué, o por qué no?

1. About how long do you think it takes, from start to finish, now that you have an appointment?

¿Cómo cuánto tiempo piensa usted que toma, desde el principio hasta el final, ahora que tiene una cita?

- 1. How long did it used to take, before you had an appointment?

¿Cuánto tiempo le tomaba antes, cuando no tenía cita?

1. *For clients with appointments in week 1:*

Currently your appointment at the food pantry is early in the month, about the same time as when your LoneStar card is refilled. What’s that like, having your food pantry appointment about the same time as when you get your LoneStar money?

Actualmente su cita a la despensa de alimentos es a principios de mes, alrededor del mismo tiempo que cuando se recarga su tarjeta de LoneStar. ¿Cómo es, tener la cita en la despensa de alimentos aproximadamente al mismo tiempo que cuando recibe su dinero de LoneStar?

*For clients with appointments in week 3:*

Currently your appointment at the food pantry is late in the month, a few weeks after your LoneStar card is refilled. What’s that like, having your food pantry appointment at a different time than when you get your LoneStar money?

Actualmente su cita a la despensa de alimentos es en los últimos días del mes, unas semanas después de cuando se recarga su tarjeta de LoneStar. ¿Cómo es, tener la cita con la despensa de alimentos en un momento diferente al de cuando recibe su dinero de LoneStar?

- 1. Do you ever worry that you might run out of food at the end of the month?

¿Alguna vez le preocupa que se pueda quedar sin alimentos al fin del mes?

- 1. Do you like having your appointment during the **1^st^/3^rd^** week of the month, or is there another time of the month when you’d prefer to have your appointment?

¿Le gusta a usted tener la cita durante **la 1ª/3ª** semana del mes, o hay otro momento del mes cuando preferiría tener su cita?

*If client likes when they have their appointment:*

What do you like about having your appointment during the ***1^st^/3^rd^*** week of the month?

¿Qué es lo que le gusta sobre tener su cita durante **1ª/3ª** semana del mes?

*If client would prefer a different time:*

What time would be better, and why?

¿Qué momento sería mejor, y por qué?

**FOOD BUYING HABITS**

**HÁBITOS DE COMPRAS DE ALIMENTOS**

Think about the last time you went to the store to purchase food.

Piense en la última vez que fue a la tienda para comprar alimentos.

1. Tell me about what you bought.

Dígame sobre lo que compró.

- 1. For example, did you go to buy food for a single meal? Or did you buy food for a single day or for a few days? Tell me about that.

Por ejemplo, ¿fue a la tienda para comprar para una sola comida? ¿O compró alimentos para un día solamente o para unos días? Dígame sobre eso.

- 1. When you go shopping for food, do you typically go home with a lot of food, or do you usually do small trips where you go home with small amounts of food? Tell me why that is.

Cuando va a comprar alimentos, ¿usted típicamente regresa a la casa con muchos alimentos, o usualmente hace pequeños mandados en donde regresa con pocas cantidades de alimentos? Dígame por qué es así.

1. How much did you think about the foods you were going to buy before you went to the store?

¿Qué tanto pensó en los alimentos que iba a comprar antes de ir a la tienda?

- 1. For example, did you make a list of what you wanted to buy before you went, or did you decide once you were inside the store? Or was it a little bit of both?

Por ejemplo, ¿hizo una lista de lo que quería comprar antes de ir, o decidió una vez que estaba en la tienda? ¿O hizo un poco de los dos?

- 1. What kinds of things affect what you buy in the store? For example, if things are on sale? If your kids are with you and they want certain things?

¿Qué tipos de cosas afectan lo que compran en la tienda? Por ejemplo, ¿si hay cosas en oferta? ¿Si sus hijos están con usted y quieren ciertas cosas?

1. When you plan ahead which things to buy, what are the factors you’re considering?

Cuando usted hace planes de qué cosas comprar, ¿cuáles son los factores que usted está considerando?

- 1. For example, did you plan how much money or LoneStar to spend before you went to the store?

Por ejemplo, ¿hizo planes de cuánto dinero o LoneStar iba a gastar antes de ir a la tienda?

- 1. Did you plan what you were going to cook that week?

¿Hizo planes de lo que iba a cocinar esa semana?

- 1. Did you consider who was going to be at your house the next few days?

¿Consideró quién iba a estar en su casa en los próximos días?

1. What type of store do you usually buy food from?

¿De qué tipo de tienda usualmente compra sus alimentos?

- 1. For example, a convenience store (like 7-11)? Regular grocery store (like Kroeger)? Dollar store? Walmart or other kind of large supercenter store?

Por ejemplo, ¿una tienda de conveniencia (como 7-11)? ¿Una tienda regular de alimentos (como Kroeger)? ¿El “Dollar Store”? ¿Walmart u otra tienda al por mayor supercentro?

1. Think about the last time you were at the store to buy food. Did you have enough money (e.g. LoneStar/EBT, cash, check, debit, or credit card) to buy the food you needed? Tell me about that.

Piense en la última vez que estuvo en la tienda para comprar alimentos. Tenía suficiente dinero (por ejemplo, LoneStar/EBT, dinero en efectivo, cheque, tarjeta de débito, o tarjeta de crédito) para comprar los alimentos que necesitaba? Dígame sobre eso.

- 1. Have you ever NOT had enough money to buy the food you needed? Tell me what that felt like.

¿Alguna vez NO tuvo suficiente dinero para comprar la comida que necesitaba? Dígame cómo se sintió eso.

I’m interested in understanding how you make decisions about what food to buy from the store and when, knowing that you also have these appointments at the food pantry.

Estoy interesada en entender cómo toma decisiones sobre qué alimentos comprar de la tienda y cuándo, sabiendo que tiene estas citas en la despensa de alimentos.

1. Do you usually shop for food at the store before or after your food pantry appointments each month? Or does it vary?

¿Normalmente compra alimentos en la tienda antes o después de su cita en la despensa cada mes? ¿O varía?

- 1. What kind of food do you buy *before* you go to the pantry? Why is that?

¿Qué tipo de alimentos compra *antes* de ir a la despensa? ¿Por qué?

- 1. What kind of food do you buy *after* you go to the pantry? Why is that?

¿Qué tipo de alimentos compra *después* de ir a la despensa? ¿Por qué?

1. Do you ever buy food from the store more than 2 weeks after your food pantry appointment? Tell me about that.

¿Alguna vez compra alimentos de la tienda más de dos semanas después de su cita en la despensa? Dígame sobre eso.

1. Tell me about the food you buy at the store. Do you buy the store food to serve *with* the pantry food to make a meal, or are they for separate meals? For example, if you got beans at the pantry, would you purchase rice at the store? Or is the store food for a separate meal entirely?

Dígame sobre los alimentos que compra en la tienda. ¿Compra alimentos de la tienda para servir *con* alimentos de la despensa y para preparar una comida, o son para comidas separadas? Por ejemplo, si recibió frijoles de la despensa, ¿compraría arroz de la tienda? ¿O los alimentos de la tienda son para comidas totalmente separadas?

Now think back to before you had appointments at the food pantry, when you would choose when and whether to go to the food pantry. I’m interested in understanding whether having these appointments has changed any of your food buying habits compared to when you didn’t have an appointment.

Ahora piense antes de cuando tenía citas en la despensa de alimentos, cuando escogería cuándo y sí ir a la despensa. Estoy interesada en entender si tener estas citas ha cambiado cualquieras de sus hábitos de compras de alimentos comparado con cuando no tenía cita.

1. For example, has having these appointments changed how you plan your meals?

Por ejemplo, ¿el tener estas citas cambió cómo planifica sus comidas?

1. Has having these appointments changed what you buy at the store?

¿El tener estas citas cambió lo que compra en la tienda?

1. Has having these appointments changed the type of stores you shop at?

¿El tener estas citas cambió el tipo de tiendas en las que compra?

1. Has having these appointments changed how much money you spend at the store?

¿El tener estas citas cambió cuánto dinero gasta en la tienda?

1. Has having these appointments changed how often you buy food?

¿El tener estas citas cambió qué tan frecuente compra alimentos?

**CORONAVIRUS**

**CORONAVIRUS**

Lastly, tell me how your personal situation has changed since the coronavirus (e.g. with employment, childcare).

Por último, dígame cómo ha cambiado su situación personal desde el coronavirus (por ejemplo, con el trabajo, el cuidado para los niños).

1. How has it affected your food needs?

¿Cómo ha afectado sus necesidades alimenticias?

1. How has it changed the way you plan your food shopping?

¿Cómo ha cambiado la manera en que planifica sus compras de alimentos?

- 1. For example, do you go to the store more or less often?

¿Por ejemplo, va a la tienda más o menos frecuente?

- 1. Has it changed the amount or what types of foods you purchase with your Lonestar card?

¿Ha cambiado la cantidad o qué tipos de alimentos compra con su tarjeta de LoneStar?

1. Are your appointments at Crossroads more or less helpful since the coronavirus? Tell me why.

¿Desde el coronavirus, son sus citas a Crossroads más o menos útiles? Dígame por qué.

1. Has your use of Crossroads’ services changed in any way during the past 2 months (e.g. need more food now that kids are not in school)?

¿Ha cambiado de cualquier manera su uso de los servicios de Crossroads durante los últimos dos meses? Por ejemplo, ¿necesita más alimentos ahora que los niños no están en la escuela?

1. Have you been able to get enough food for your family during the last 2 months, or have there been times when you didn’t have enough food?

¿Ha podido conseguir suficiente comida para su familia durante los últimos 2 meses, o ha habido momentos cuando no tuvo suficiente comida?

1. How does this compare to before the coronavirus?

¿Cómo se compara esto a antes del coronavirus?
